# Effect of temperature – humidity index on the onset of post- partum ovarian activity and reproductive behavior in *Bos indicus* cows

**DOI:** 10.21451/1984-3143-AR2019-0074

**Published:** 2020-02-20

**Authors:** Ramiro Fernando Díaz, Carlos Salvador Galina, Emilio Manuel Aranda, Lorenzo Armando Aceves, Jaime Gallegos Sánchez, Jose Luis Pablos

**Affiliations:** 1 Instituto de Investigaciones en Biomedicina, Universidad San Francisco de Quito, Quito, Ecuador; 2 Escuela de Medicina Veterinaria, Universidad San Francisco de Quito, Quito, Ecuador; 3 Departamento de Reproducción, Facultad de Medicina Veterinaria y Zootecnia, Universidad Nacional Autónoma de México, Ciudad de México, México; 4 Colegio de Postgraduados, Campus Tabasco, Tabasco, México; 5 Colegio de Postgraduados, Campus Montecillo, Montecillo, México; 6 Departamento de Genética y Bioestadística, Facultad de Medicina Veterinaria y Zootecnia, Universidad Nacional Autónoma de México, Ciudad de México, México

**Keywords:** beef cows, fat thickness, natural mating, temperature-humidity index, reproductive cyclicity

## Abstract

The effect of climatic factors on ovarian activity and reproductive behavior (RB) was evaluated in 46 *Bos indicus* cows kept under grazing conditions. Temperature-humidity index (THI) was used as an indicator of stress and divided in alert, damage and emergency levels. Fat thickness (FAT) was taken during the last trimester of gestation (LTG) to approximately 90d postpartum (PP). At 30d PP animals received a progesterone (P_4_)-releasing device (CIDR) which was withdrawn 9d later. Ovarian activity was assessed by blood progesterone on days 21, 24, 27, 30, 49, 51, and 54 PP. Animals were divided into three groups, higher, and moderate RB and non-behavior. Sixty percent presented a THI >74 increasing dramatically from June to September up to >78. During LTG, animals lost 27% of their body reserves contrasting to PP where an increase of 2.6% (P=0.002) was observed. The percentages of cyclic and non-cyclic animals were 57 and 43%, respectively (P> 0.05). Seventy-two percent displayed RB and 28% were non-behavior (P<0.05). A negative correlation (r = -0.307; P = 0.038) between THI and RB, and a positive correlation (r = 0.427; P = 0.003) between the onset of ovarian activity and RB were observed. Differences in THI during the LTG (P<0.01) were observed between cyclic and non-cyclic animals. Non-behavior cows in the LTG had a higher THI (P <0.05). High levels of THI have a negative effect on the resumption of ovarian activity and RB in *Bos indicus* especially if high THI occurs during the last trimester of gestation.

## Introduction

The direct effect of climate on livestock production in the tropics has been studied for a long time (Gwazdauskas, 1985; Hansen, 2007). The detrimental effects of high environmental temperature include poor oocyte and embryo quality, reduced dominance and steroidogenic capacity of the selected follicle, decreased uterine blood flow and endometrial dysfunction (Roth, 2008). The temperature-humidity index (THI) has been widely used as an indicator of thermal stress in livestock (Hansen, 2007), forming the basis of the Livestock Weather Safety Index (Livestock Conservation Incorporated, 1970). THI above 74 has been targeted as an indicator of climatic stress for cattle (Arias et al., 2008). There is also evidence for a negative effect of a high THI on conception rate, in the latter, lower levels have been recorded during the summer compared to the winter (Villa-Mancera et al., 2011).

High environmental temperatures bring about a decrease in feed intake and play a decisive role in the management of cattle (Renaudeau et al., 2012). The dichotomy between periods of dry weather with scarce forage and the rainy season where fodder is more plentiful complicates the handling and maintenance of cattle (Grings et al., 2005). This situation has been made worse recently by climate change (Myers et al., 2017) as the variability of the rainy season coupled with overgrazing, jeopardize husbandry procedures particularly for animals in the tropics (Raiten and Aimone, 2016).

Fat thickness (FAT) has been shown to be a reliable indicator of the level of energy reserves in the animal (Schröder and Staufenbiel, 2006). There is evidence to suggest that FAT during the last trimester of gestation, is related to reproductive ability (Wettemann et al., 2003). Thus, early resumption of ovarian activity is closely linked to the capacity of the dam to recover from negative energy balance caused by stress during parturition (Montiel and Ahuja, 2005) and possibly from heat stress (Alves et al., 2014).

The objective of the present study is, therefore, to evaluate using THI as an indicator of heat stress on resumption of ovarian activity and reproductive behavior of *Bos indicus* cows.

## Methods

### Ethical statement

The Animal Care Internal Committee (CICUA) of the Faculty of Veterinary Medicine and Zootechnics of the National Autonomous University of Mexico approved the methods used during the present work in accordance with The Code of Ethics of the World Medical Association (Declaration of Helsinki) with the approval number: DC-2015/2-8.

### Location

This study was carried out in the research station belonging to the Colegio de Postgraduados (COLPOS), located in the State of Tabasco, Mexico, at 93° 35' 1.31” W and 17° 59' 12.58” N. The climate is tropical and humid in the absence of a defined dry season (Ruiz-Álvarez et al., 2012).

The temperature-humidity index (THI) was calculated taking the average environmental temperature and relative humidity of the period in which the study was conducted, according to the formula proposed by García-Ispierto et al. (2007):

$THI=\;\left(0.8\;x\;T+\;\left(RH/100\right)\;x\;\left(T\;-14.4\right)\;+\;46.4\right)$ (1)

Where: *THI,* is the temperature-humidity index; *T*, is the mean temperature expressed in °C; and *RH*, is the average relative humidity expressed in percentage. Thus, the maximum and minimum ranges of THI levels were calculated. In addition, the number of hours over 74, 78 and 80 of THI for each day between the months of January and September of 2016, were considered as indicators of heat stress in alert, damage, and emergency levels, respectively (Arias et al., 2008). In turn, the THI of each animal was measured 56 days before calving and 56 days postpartum, day zero was the calving date. In addition, three days after the summer solstice (June 24), rectal temperature was measured in a subsample of 14 animals chosen at random. These measurements were taken every 10 minutes from 10:00 until 14:00 hrs.

### Animals

Forty-six *Bos indicus* crossbred animals in their last trimester of gestation and raised under an intensive grazing system, were used. Pastures were mainly a combination of *Cynodon nlemfuensis*, *Paspalum virgatum l.*, *Paspalum fasciculatum willd*, *Paspalum conjugatum*, *Desmodium adscendens.* No supplementary feeding was given during this period, although they had access *ad libitum* to water and mineral salts. Due to the nature of the study, it was not feasible to take samples of either forage or consumption.

### Fat thickness and body condition score

All measurements of fat thickness (FAT), expressed in centimeters (cm), were taken two weeks apart, from the beginning of the last trimester of gestation to 90 ± 6 d PP, using the ALOKA ProSound 2 ultrasound equipment with a convexed array transducer 3.5 MHz. The FAT was measured in the thurl area located midway between the tuber coxae (hooks) and the tuber ischiae (pins), 2 to 3 cm above the greater trochanter of the femur (Schröder and Staufenbiel, 2006). Body condition score was evaluated at the onset of the study by observation using the scale 1 to 9 (1= extremely thin, 9= very fat) as indicated by Wagner et al. (1988).

### Resumption of postpartum ovarian activity and reproductive behavior.

**Figure 1:**
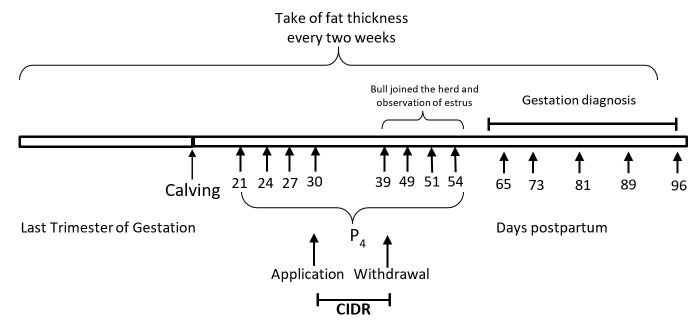
Timeline of specific experimental events with *Bos indicus* crossbred cows. P_4_: Day postpartum when the blood sample was taken to measure concentrations of progesterone.

All animals with a confidence interval of 90% around a mean of 30 days postpartum, received a progesterone-releasing device (Eazy-breed CIDR™, 1.9 g of natural progesterone in silicone, Zoetis®, México) which was withdrawn 9 days later followed by an injection of 25mg of Prostaglandin F2α (Dinoprost, Lutalyse, Zoetis®, México). At the time of CIDR withdrawal, animals were exposed to two bulls following a breeding soundness evaluation (BSE). Onset of ovarian activity was assessed by blood levels of progesterone, which were taken on days 21, 24, 27, 30, 59, 51 and 54 postpartum (Figure 1). Progesterone concentrations were determined by a solid phase radioimmunoassay in 100 µl of serum using commercial kits (Pharmaceuticals, Diagnostic Division). The intra and inter-assay coefficients of variation were 7.4% and 6.9%, respectively. A progesterone concentration above 1 ng/ml in two or more successive samples was indicative of luteal ovarian activity (Pulido et al., 1991). The cows were then divided in two groups: cyclic and non-cyclic.

All animals were continuously observed for 12 hours (6:00 to 18:00) for 3 days after CIDR withdrawal to characterize behavioral changes of the females in the presence of the bull (Molina et al., 2002). ESTROTECT patches (Rockway Inc., Spring Valley, WI, USA) were placed on all cows to identify those that were in estrus after 18:00 h. Changes in the color of the patches were registered every morning at 06:00h, classifying them in activated, inconclusive and non- activated patches as proposed by Talukder et al. (2014). Signs of reproductive behavior (RB) were classified as: sniffing, licking and flehmen, (courtship activity); attempt to mount and mounts or allowed mounting, (estrual activity). Therefore, animals were divided in three groups: Estrual Activity, those that showed signs of estrus (mount or attempt to mount). Courtship activity, those that showed signs of courtship (sniffing, licking and flehmen); and those animals not displaying reproductive behavior during the experiment (non-behavior; Orihuela et al., 1983). Pregnancy diagnosis was carried in those cows with more than 28 days of exposure to the bull on a weekly basis up to 90 ± 6 d PP, using an ultrasound ALOKA ProSound 2 equipped with linear transducer of 7.5 MHz.

### Statistical analyses

Possible differences in FAT during the last trimester of gestation and the postpartum were analyzed using Student “t” test. In turn, total relative change in back fat (ΔFAT= [initial measure - final measure] / initial measure) was determined. To analyze the changes in the measurements of rectal temperature at various times of the day, a one-way ANOVA was performed. Variations of FAT between groups were evaluated by ANOVA for repeated measures followed by Bonferroni adjustments for multiple comparisons. The proportions of the ovarian activity and reproductive behavior were evaluated with a 95% confidence interval with the support of a Chi-square analysis. A correlation coefficient was used to determine the relationship between THI, onset of ovarian activity and reproductive behavior. In FAT the effect of the cyclic and non-cyclic groups, groups according to the reproductive behavior (high, moderate or no behavior) and the day in which the FAT was measured by means of the Generalized Estimating Equations model (GEE) was evaluated. The estimators of the model effects that were selected were those with the lowest standard error according to the assumed structure of the model errors. In this case we considered the structures of independence, autoregressive of the first order (AR1), interchangeable and without structure (Song, 2007). All statistical analyses were performed on IBM SPSS 22 and JMP 6.0 statistical packages. P-values <0.05 were statistically different.

## Results

### Temperature-humidity Index (THI)

For the whole duration of the experimental period, 60% presented a THI> 74, that is, levels related to a stressful environment and confirmed by calculating the number of hours presented with a THI> 74,> 78 and > 80 in the same period (Table 1). For example, during the month of January, the range of hours >74 was 0-13 h, with up to 7h with values >78 and up to 4h with a THI above 80. In relation to the subsample of 14 animals whose rectal temperature was taken intensively, during mid-morning, the animals presented an average temperature of 38.73 ^o^C with a range between 37.5 and 39.9 ^o^C. At noon, as expected, the temperature rose to 40.04 ^o^C (P=0.07) with a range of 38.3 to 45 degrees. At 14:00 hrs., the average temperature was 41.14 ^o^C (P = 0.001 for the first and P = 0.126 for the second measurement) with a range of 38.5 to 46 degrees.

**Table 1:** Number of hours (mean; standard deviation (SD) and range) where the THI was above 74, 78 and 80 indicating animals were exposed to a stressful environment.

**Month**	**THI > 74**			**THI > 78**			**THI > 80**		
	**Mean**	**SD**	**range**	**Mean**	**SD**	**Range**	**Mean**	**SD**	**Range**
January	3.3	4.6	0-13	1.2	2.2	0-7	0.5	1.2	0-4
February	2.4	3.3	0-12	0.4	1.5	0-7	0.2	1.0	0-5
March	11.1	4.6	1-19	5.7	4.1	0-13	3.7	3.2	0-10
April	13.5	6.6	0-19	7.1	4.5	0-14	4.7	4.0	0-11
May	15.3	4.0	7-22	9.0	4.1	0-15	6.0	3.9	0-12
June	15.2	2.4	7-20	8.1	2.5	1-12	4.7	2.3	0-8
July	14.3	2.1	5-16	7.6	1.7	2-10	4.2	1.7	0-7
August	14.4	2.3	5-19	6.8	2.2	3-10	3.2	2.2	0-7
September	13.5	2.1	5-16	6.1	2.1	3-8	2.7	1.8	0-5

### Fat Thickness (FAT)

The body condition score at the start of the experiment was on average 3.8 ± 1.33 while animals at the start and the end of the last trimester of gestation (LTG) had an average FAT of 0.197 ± 0.06 and 0.198 ± 0.07 cm, respectively. In this period, the relative change in fat indicates that the animals lost 27% of their reserves. In the postpartum, animals started with an average of 0.21 ± 0.05 and ended with 0.22 ± 0.04 cm. The relative change of fat in this period indicates an increase of 2.6%. A significant difference was found between the relative variations of dorsal fat during the LTG and the postpartum period (P = 0.002).

### Resumption of postpartum ovarian activity and Reproductive Behavior (RB)

The percentages of cyclic and non-cyclic animals during the experiment were 57% (26/46) and 43% (20/46) respectively (P> 0.05). Thus, 27% (7/26) were cycling at 21d PP; 27% (7/26) had ovarian activity between 21 and 30d PP; 27% (7/26) between 30 and 40d PP; and 19% (5/26) cycled after 40d PP (P>0.05).

**Figure 2:**
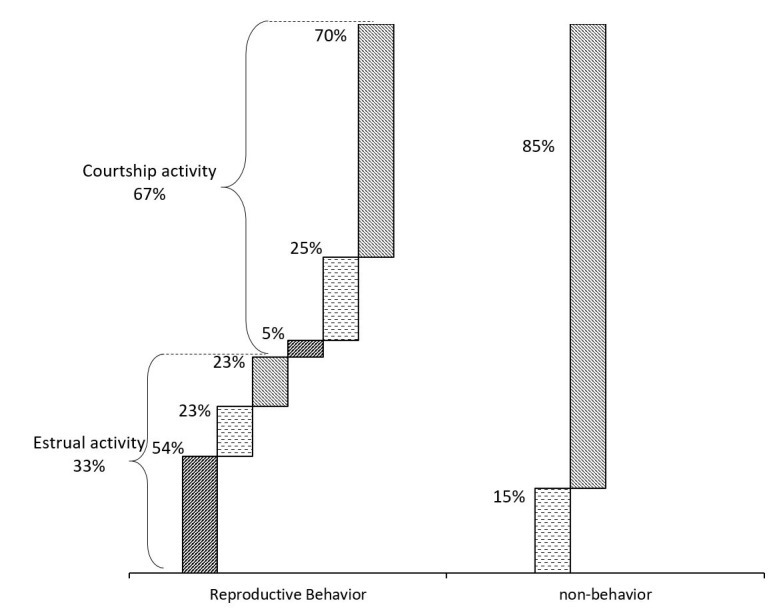
Proportions of different marks in the patches used in cattle Bos indicus to indicate their reproductive behavior (RB). Values expressed in percentages.

On the other hand, 72% (33/46) of the animals displayed mostly courtship activities and 28% (13/46) were non-behavior (P<0.05). Of the animals expressing reproductive behavior, 33% (11/33) had estrual activity, whereas the remaining 67% (22/33) showed courtship activity (P>0.05). Figure 2 shows the percentage of different marks in the patches used in observing RB. Animals with estrual activity have the highest proportion of activated patches; conversely, non-behavior animals have the highest proportion of non- activated patches.

Thirty percent of the animals observed with reproductive behavior, expressed mounting activity, of these, 60% had a skin patch, 30% and 10%, dubious and intact patches, respectively. Ultimately, the pregnancy rate in the present study, was 22% (10/46). By calculating only, the number of cows at risk of becoming pregnant, i.e. cycling, the conception rate was 38% (10/26). Of all the pregnant animals displaying mounting behavior and associated signs of estrus, 70% and 30% presented higher and moderate RB, respectively (P<0.05). Moreover, all initiated their ovarian activity as evidenced by high progesterone levels.

### Relationship between temperature-humidity index, onset of ovarian activity, reproductive behavior and changes in FAT

**Figure 3:**
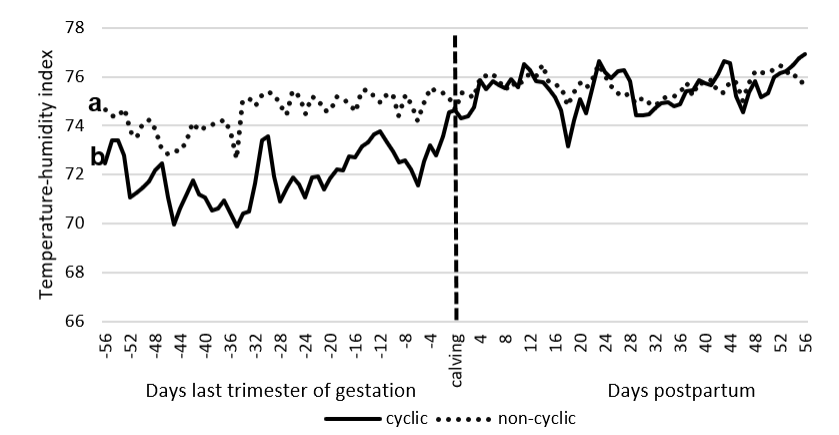
THI levels during the last trimester of gestation and postpartum of animals that cycled (57%) and non-cycling (43%) between the months of January and September of 2016. a, b: significant difference during the last trimester of gestation (P <0.01). In the postpartum period, there was no significant difference (P>0.05).

A low negative correlation (r = -0.307; P = 0.038) was found between the mean THI and reproductive behavior, and a moderate positive correlation (r = 0.427; P = 0.003) between the onset of ovarian activity and reproductive behavior. Conversely, the THI and the onset of cyclicity had a very low and non-significant correlation (r = -0.058; P = 0.702). Figure 3 depicts the levels of THI during the last trimester of gestation and the postpartum period in cyclic and non-cyclic animals. A significant difference was observed during the last trimester of gestation (P<0.01) and seemingly, the proportion of animals starting their cyclicity decreased as THI increased. In addition, although not significant, the period postpartum of the cows in the experiment, appears to be affected by an increase of THI.

At the onset of the LTG, the cyclic and non-cyclic animals had FAT values of 0.21 ± 0.07 vs. 0.19 ± 0.04 cm.

GEE analysis indicates that in Fat there is a day effect (P = 0.0001) in which the FAT was evaluated, and in the RB groups (P = 0.0001), but no effect was observed of the cyclic and non-cyclic groups (P = 0.89). An interaction was observed of day vs. cyclic and non-cyclic groups (P = 0.0001), Day vs. RB groups (P = 0.039), and cyclic and non-cyclic groups vs. RB groups (P = 0.039); while the effect of the covariable THI was significant (P = 0.0001).

**Figure 4:**
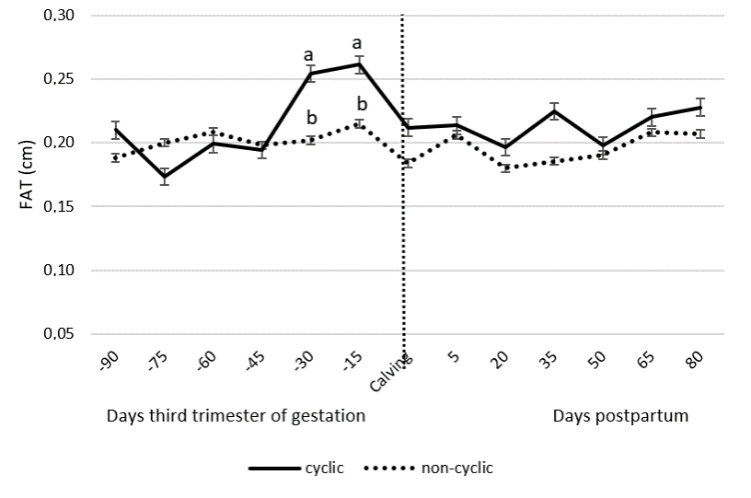
FAT during the last trimester of gestation and postpartum of cyclic and non- cyclic cows. Different letters indicate statistical difference (P <0.05) between groups.

FAT in cyclic animals decreased in the last trimester of gestation with a variation of 37% but increased in the postpartum with a variation of 5% (P=0.002). On the other hand, in the non-cyclic animals, FAT decreased both in the last trimester of gestation and in the postpartum period, (P = 0.315, Figure 4).

**Figure 5:**
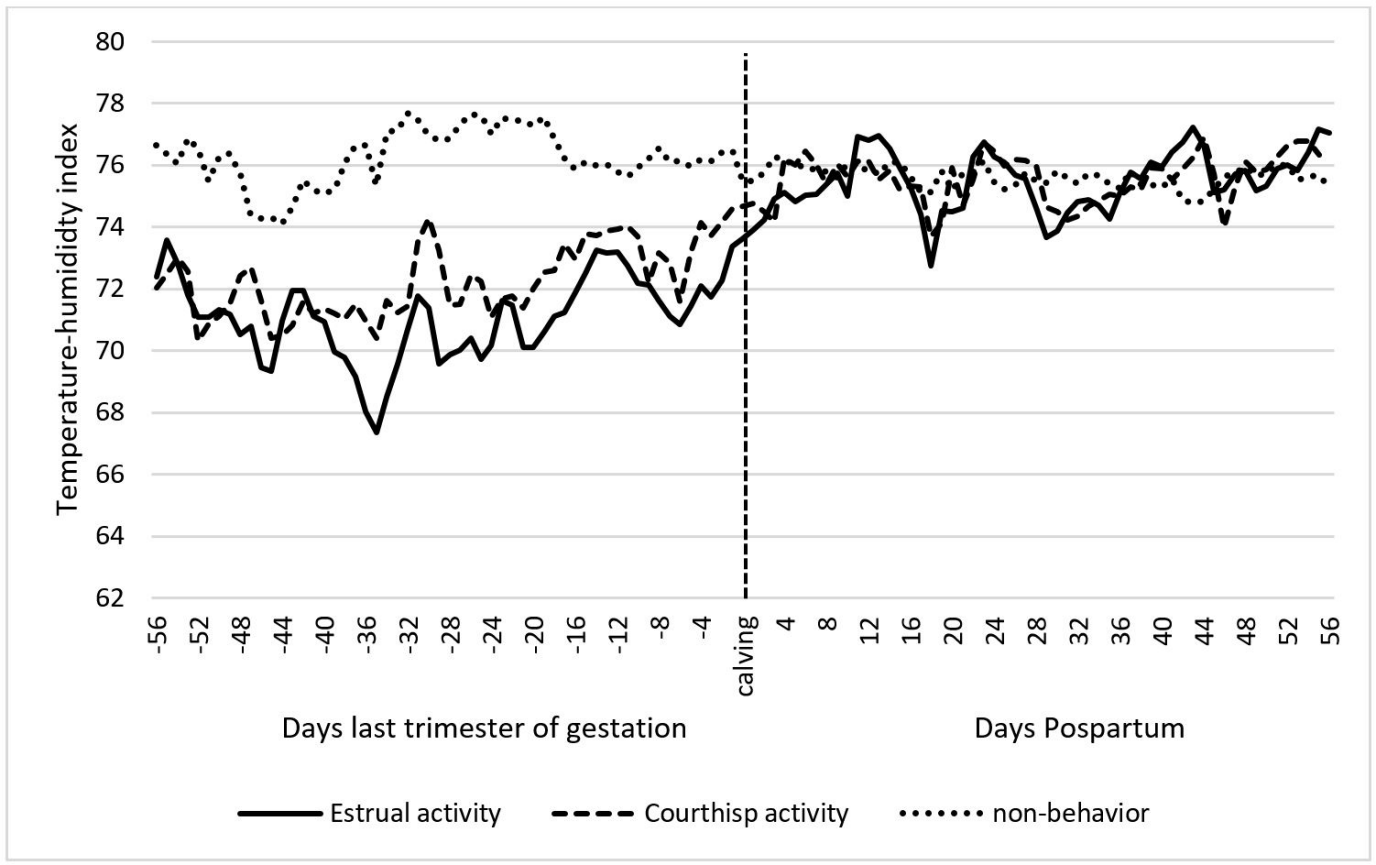
THI levels in animals with Estrual Activity, Courtship activity and no reproductive behavior between the months of January and September of 2016. Different letters indicate statistical difference (P <0.05) in the last trimester of gestation.

Likewise, during the LTG, animals that did not display reproductive behavior (non-behavior) had a higher THI (P <0.05) compared to animals with a higher or moderate RB (Figure 5). FAT presents significant differences (P <0.001) between the groups that presented some reproductive behavior and the group that did not show any reproductive behavior.

## Discussion

The temperature-humidity index was > 74 for 60% of the time possibly indicating that stressful levels prevailed for almost 2/3 of the duration of the present investigation. However, between the months of May to September, at no time was THI <74 and at a maximum in the months of March until May (Table 1). This was reflected in the subsample of 14 animals whose temperature on average rose almost three degrees. These observations are similar to those reported in other studies indicating that the state of Tabasco has a climate where high temperature and humidity levels predominate throughout the year (Angulo-Córdova et al., 2004). Thus, a THI above the limit of comfort (> 74) can affect the reproductive ability of the animals (Amundson et al., 2006) causing a decrease in the normal development of cattle (da Costa et al., 2015). Moreover, a high THI can affect feed intake in animals (Renaudeau et al., 2012) which would explain the poor body condition at the start of the experiment (3.8 ± 1.33) and reflected in back fat loss during the last trimester of gestation. A small gain of body fat was observed during the postpartum (2.6%). Possibly, adaptive mechanisms such this one are required for recovery from the physiological process of calving, could present a major hurdle to re-establishing normal homeostasis. Indeed, physiological adaptation processes exist as mechanisms of thermoregulation (e.g. increased respiratory rate) allowing animals under conditions of stressful THI levels to feed and recover their energy reserves (Renaudeau et al., 2012).

The percentage of animals starting their cyclicity was 57%. This proportion is low compared to previous reports where values fluctuated between 70 to 92% (Pérez-Torres et al., 2015; Mondragón et al., 2016). On the other hand, 43% of animals did not ovulate. Nonetheless, this percentage is higher than that reported in previous studies where this figure does not exceed 29% (Pérez- Torres et al., 2015). The discrepancies between values could be the result of differences in the high levels of THI that animals were able to tolerate. Unfortunately, having the animals at pasture did not allow us to consider taking accurate measurements of stress, such as respiratory rate and body temperature. More studies are in demand if THI is an appropriate indicator of stress under the lowland tropics, where humidity and solar radiation could affect the interpretation of stress. Decreased steroidogenic capacity of the dominant follicle is one of the characteristics of the negative effects of heat stress (Roth, 2008). This may decrease their ability to generate a pre-ovulatory peak of luteinizing hormone (LH), ovulate and form a corpus luteum. At the same time, animals without ovarian activity tolerated higher THI levels during the last trimester of gestation compared to those cycling (P <0.01, Figure 3). Possibly the effect of even higher THI in the last trimester of gestation, may have affected the resumption of ovarian activity. Some authors suggest that heat stress alters follicular development by reducing size in both the first and the second wave (Badinga et al., 1993). At the same time, it attenuates the dominance by increasing the number of large follicles (Wolfenson et al., 1995). In the present experiment, the follicular diameter of the animals was not evaluated, but it could be speculated that the effects of a higher THI during the last trimester of gestation annulled the resumption of ovarian activity (Hansen, 2007). This could be confirmed by observing if the proportion of animals starting their cyclicity remains constant during postpartum when THI is also unchanging. More research is needed on this topic.

Cows that cycled before 21d PP appear to be a constant feature in Zebu type cattle, in effect previous studies have shown a similar trend (Pérez-Torres et al., 2015; Mondragón et al., 2016). The first dominant follicle in *Bos indicus* appears from 10 to 15d PP (Murphy et al., 1990; Crowe et al., 2014) but ovulation does not occur until approximately 30d PP (Murphy et al., 1990). A similar trend in ovulation was found in the present study.

On the other hand, the group displaying ovarian activity showed greater variation in body fat compared to non-cyclic cows (Figure 4). Whilst in the cyclic group, variations occurred both during the last trimester of gestation (down 37%) and in the postpartum (increase of 5%; P = 0.002), in the non-cyclic group, body fat reserves were not statistically modified (P = 0.315). In a previous study, non-cyclic cows showed a decrease in dorsal fat from the last trimester of gestation to the postpartum period, while animals with luteal activity maintained their energy reserves over these two periods (Díaz et al., 2017). This apparent contradiction between the results of the two studies is possibly due to the level of FAT reserves in the animals. While in the present investigation the FAT levels did not exceed 0.21 cm, in other studies, animals had variations of body fat up to 0.40 cm. This means that the animals in this experiment had very low levels of fat and possibly only have what is needed to maintain basic vital processes. Only those able to mobilize their energy reserves could trigger their ovarian activity.

In the present experiment, 72% of animals showed some reproductive behavior (RB), of these, only 33% expressed estrus activity. This means that of the total of animals only 24% expressed definitive signs of estrus (attempt to mount and mount). This percentage is quite low compared to that reported by other authors where the proportion of animals in estrus represented around 75% (Maquivar et al., 2002; Acevedo et al., 2007). This difference could be explained by the climatic conditions in which the animals were found. There is evidence of the negative effects of caloric stress on reproduction (Hansen, 2007, 2009). The animals that did not show any reproductive behavior were under much stronger heat stress during the last third of gestation than those who did show some behavior (Figure 5).

Pregnancy rate was 38% and low compared to other studies in the tropics with rates over 70% (Escrivão et al., 2012). However, animals in the previous studies, probably did not experience the harsh environmental conditions of the present experiment. Interestingly, some animals with moderate reproductive behavior became pregnant but to a lesser extent than animals with high RB. The expression of estrous behavior is characteristic of animals that have started cyclicity and therefore, it is understandable that they are more likely to become pregnant (for review see Galina and Orihuela, 2007).

Furthermore, 28% of all animals did not present RB (non-behavior). During the last trimester of gestation, these animals were under higher THI levels than cows with higher and moderate RB (P <0.05). A reduction in steroidogenic capacity of follicles under thermal stress is characterized by less aromatase activity of granulosa cells and decreased estradiol concentration in the fluid of the dominant follicle (Badinga et al., 1993). Among the potentially adverse effects of heat stress associated with low estradiol levels, are reduced plasma estradiol concentration (Badinga et al., 1993; Wolfenson et al., 1995) impairing estrus duration and intensity, thus reducing the number of mounts (Gwazdauskas et al., 1981) as observed in the present investigation.

In conclusion, high levels of THI have a negative effect on the resumption of ovarian activity postpartum and expression of reproductive behavior in *Bos indicus* cows, especially when high THI occurs during the last trimester of gestation.
